# Clinical Outcomes of COVID-19 Infection among Patients with Chronic Obstructive Pulmonary Disease: Findings from the Philippine CORONA Study

**DOI:** 10.3390/clinpract13060124

**Published:** 2023-11-10

**Authors:** Roland Dominic G. Jamora, Albert B. Albay, Mary Bianca Doreen F. Ditching, Marie Charmaine C. Sy, Emilio Q. Villanueva, Adrian I. Espiritu, Veeda Michelle M. Anlacan

**Affiliations:** 1 Department of Neurosciences, College of Medicine and Philippine General Hospital, University of the Philippines Manila, Manila 1000, Philippines; charmysy@gmail.com (M.C.C.S.); aiespiritu@up.edu.ph (A.I.E.); vmanlacan@up.edu.ph (V.M.M.A.); 2 Institute for Neurosciences, St. Luke’s Medical Center, Global City, Taguig 1634, Philippines; 3 Division of Pulmonary Medicine, Department of Medicine, College of Medicine and Philippine General Hospital, University of the Philippines Manila, Manila 1000, Philippines; abalbay1@up.edu.ph (A.B.A.J.); biancaditchingmd@gmail.com (M.B.D.F.D.); 4 Department of Pathology, College of Medicine and Philippine General Hospital, University of the Philippines Manila, Manila 1000, Philippines; eqvillanueva@up.edu.ph; 5 Department of Clinical Epidemiology, College of Medicine and Philippine General Hospital, University of the Philippines Manila, Manila 1000, Philippines

**Keywords:** COVID-19, COPD, smoking, mortality, respiratory failure

## Abstract

Background: The global pandemic caused by the coronavirus disease 2019 (COVID-19) resulted in many deaths from fulminant respiratory failure. Chronic obstructive pulmonary disease (COPD) is the leading cause of morbidity and mortality worldwide. There has been great concern regarding the impact of COPD on the COVID-19 illness. Methods: Data from the Philippine CORONA study were analyzed to determine the association of COPD and COVID-19 in terms of mortality, disease severity, respiratory failure, mechanical ventilation, and lengths of stay in the intensive care unit (ICU) and hospital. Results: A total of 10,881 patients were included in this study, and 156 (1.4%) patients had been diagnosed with COPD. A majority of COVID-19 patients with COPD had other existing comorbidities: hypertension, diabetes mellitus, chronic cardiac disease, and chronic kidney disease. COPD patients were 2.0× more likely to present with severe to critical COVID-19 disease. COVID-19 patients with COPD in our study have a 1.7× increased mortality, 1.6× increased respiratory failure, and 2.0× increased risk for ICU admission. Smokers with COVID-19 were 1.8× more likely to present with more severe disease and have a 1.9× increased mortality. Conclusion: Our study supports the growing evidence that COPD among COVID-19 patients is a risk factor for higher mortality, more severe form of COVID-19, higher ICU admission, and higher respiratory failure needing ventilatory support.

## 1. Introduction

The severe acute respiratory syndrome coronavirus 2 (SARS-CoV-2) has been responsible for the global coronavirus disease 2019 (COVID-19) pandemic. This highly transmissible disease can cause a wide range of symptoms from mild viral illness to fulminant respiratory failure and death [1]. As of September 2023, there has been 695,358,466 cases worldwide and 6,915,923 deaths [2]. 

Multiple descriptive studies have been made regarding the effect of pulmonary co-morbidities on the COVID 19 disease severity and outcomes [3,4,5,6,7,8]. At least one co-morbidity is present in 20–51% of COVID-19 patients, including hypertension and diabetes [3]. In the Philippines, several papers have reported on the presence of co-morbidities conferred poorer clinical outcomes (increased risk of mortality, respiratory failure, and the need for ICU admission) among Filipino COVID-19 patients [9,10,11]. 

The Global Initiative for Chronic Obstructive Lung Disease in its 2023 document defines chronic obstructive pulmonary disease (COPD) as a heterogeneous lung condition with long term respiratory symptoms due to abnormalities in the airways and/or alveoli that results in persistent, often progressive, airflow limitation [12]. To date, smoking tobacco remains the main environmental exposure that leads to the pathology of COPD. COPD is the leading cause of morbidity and mortality worldwide [12].

As COVID-19 is a predominantly respiratory infection, there has been great concern regarding patients with COPD and their susceptibility in acquiring the SARS-CoV-2 virus and risk for developing severe disease. It is an established fact that viral infections can cause COPD exacerbations that can lead to poor clinical outcomes. 

There is growing evidence on the impact of COPD as a co-morbidity in COVID-19 infection worldwide. To date there have been no published data from the Philippine population. This study aimed to determine and compare the outcomes of COVID-19 patients with COPD co-morbidity against those without in terms of COVID-19 severity, respiratory failure, ICU admission, and length of ICU stay. 

## 2. Materials and Methods

### 2.1. Study Design, Data Collection, Sampling, and Definition of Cohorts

We performed an analysis of data from the Philippine CORONA (COVID-19 Outcomes: a Retrospective Study of Neurological Manifestations and Associated Symptoms) Study with and without COPD co-morbidity. The Philippine CORONA Study is a nationwide, multicenter, comparative, retrospective, cohort study involving admitted patients with COVID-19 [13]. This was the largest Philippine study of COVID-19 to date involving 10,881 patients. COVID-19 cases were identified from the census of all participating institutions. Pertinent data were obtained through a review of medical records and encoded using electronic data collection form using Epi Info Software (V.7.2.2.16). The original cohort was approved by the individual institutional review and research boards of the hospital sites and the Single Joint Research Ethics Board of the Department of Health of the Philippines (see complete list below). 

### 2.2. Outcome Variables

In this study, the following effect of COPD on the following outcomes were measured: (a) mortality, (b) the severity of COVID-19 based on Philippine COVID-19 Living Guidelines [14] (defined as the worst COVID classification of severity throughout the admission: mild–moderate—presence of mild pneumonia or absence of pneumonia; severe—presence of dyspnea, respiratory rate above 30 breaths per minute, oxygen saturations < 93% or more than 50% lung involvement on radiologic imaging within 24–48 h; critical—presence of respiratory failure, shock, or multiorgan dysfunction), (c) respiratory failure defined as the use of ventilatory support, (d) intensive care unit (ICU) admission, (e) the length of ICU stay, (f) the length of hospital stay, and (g) ventilator days for those placed on mechanical ventilation. In this analysis, we also explored the association of smoking with COVID-19 severity and mortality. 

### 2.3. Statistical Analysis

The participants’ baseline characteristics and clinical outcomes were presented using descriptive statistics. Numerical variables that were normally distributed as assessed by the Shapiro–Wilk test for normality were presented as mean and standard deviation and as median and interquartile range (IQR), if otherwise. Categorical variables were described as count and proportion. Comparative analysis of baseline characteristics and clinical outcomes were performed between two groups: with COPD and without COPD. Student’s *t* test for variables was used to determine significant differences in the mean/median/mean rank of the different numerical variables between the two groups for normally distributed data, while the Mann–Whitney U test was performed for non-normally distributed variables. The heterogeneity of the proportions of the different categorical variables between the two groups were determined by chi-square test or Fisher exact test.

The associations between COPD and the different dichotomous and count outcome variables of interest were determined by binary logistic and Poisson regression, respectively. The logistic and Poisson regression models were adjusted for the age, sex, history of smoking, and comorbidities. In addition, the Poisson regression model was adjusted for the effect-measure modifier COVID-19 severity at nadir for lengths of hospital and ICU stay. Survival analysis was performed for the time-to-event data of mortality, respiratory failure, and admission to the intensive care unit (ICU). The time-to-event were right-censored on time-to-discharge as the exit from the time-at-risk among those who have not experienced the event, i.e., mortality, respiratory failure, or admission to ICU during their hospital stay. The associations between having COPD and the different time-to-event outcome variables of interest, adjusting for the age, sex, history of smoking, and comorbidities, were determined by a log-rank test of equality of survivor function. A cutoff of *p*-value < 0.05 identifies COPD and smoking status as significant predictors of the different outcomes of interest. Kaplan–Meier curves were constructed to visualize the survival curves of COPD versus non-COPD patients for the different time-to-event outcome variables.

## 3. Results

A total of 10,881 patients were included in the study, with 156 (1.4%) patients having been diagnosed with COPD. Table 1 presents the clinicodemographic characteristics of patients included in the study. COVID-19 patients with COPD were from the older age group 60 years old and above (78.8%, *p* < 0.001), predominantly male (84.0%, *p* < 0.001) and had a significant smoking history (52.5%, *p* < 0.001). A majority of COVID-19 patients with COPD had other existing co-morbidities. The most common of which were hypertension (66.7%, *p* < 0.001), diabetes mellitus (39.1%, *p* < 0.001), chronic cardiac disease (18.6%, *p* < 0.001), and chronic kidney disease (10.9%, *p* = 0.004). Many of the COVID-19 patients with COPD reported a history of a previous stroke (6.4% *p* = 0.026) and of neurodegenerative disorders (1.9%, *p* = 0.025).

COVID-19 patients with COPD presented more often with fever, cough, dyspnea, and sputum production compared to those without COPD diagnosis. They also tend to present with new onset neurologic symptoms manifesting as altered mental state (8.3%, *p* = 0.035) or encephalopathy (12.1%, *p* = 0.001). 

The COVID-19 patients with COPD in our study received a higher proportion of COVID-19 treatments namely glucocorticoids (55.8%, *p* < 0.001), tocilizumab (15.4%, *p* = 0.011), anti-virals (29.5%, *p* < 0.001), anti-bacterials (96.2%, *p* < 0.001), and other therapies (48.1%, *p* = 0.001) compared to those without COPD. 

**Figure 1:**
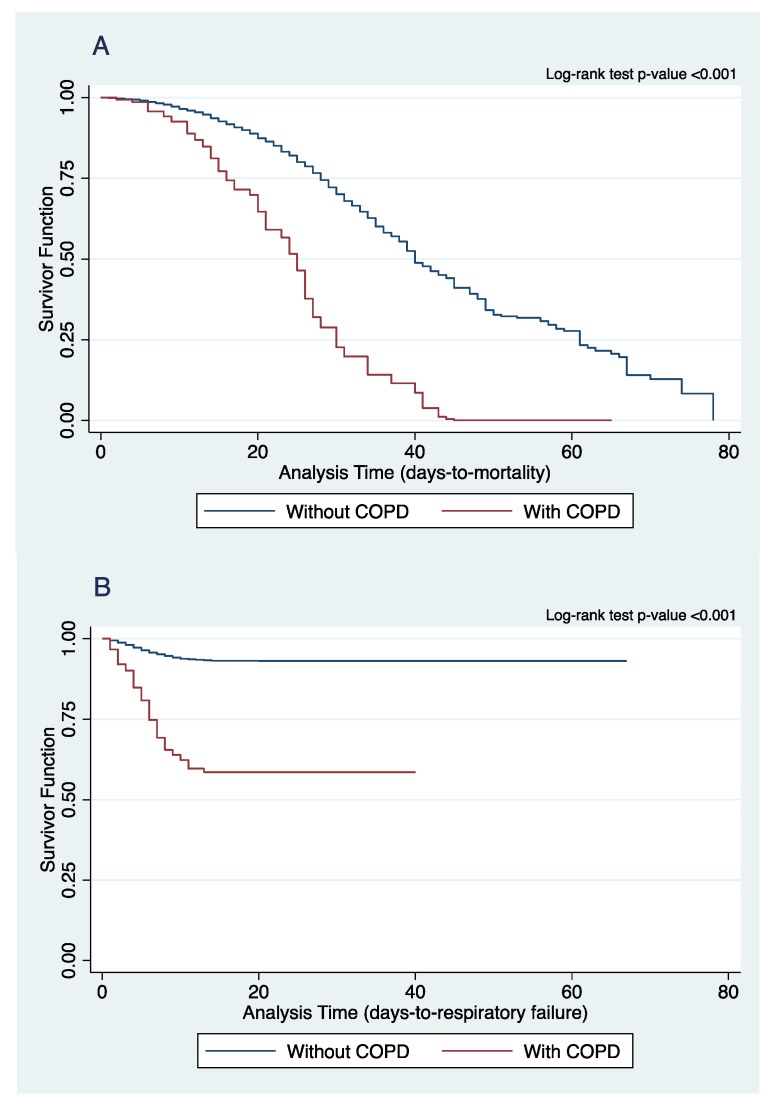

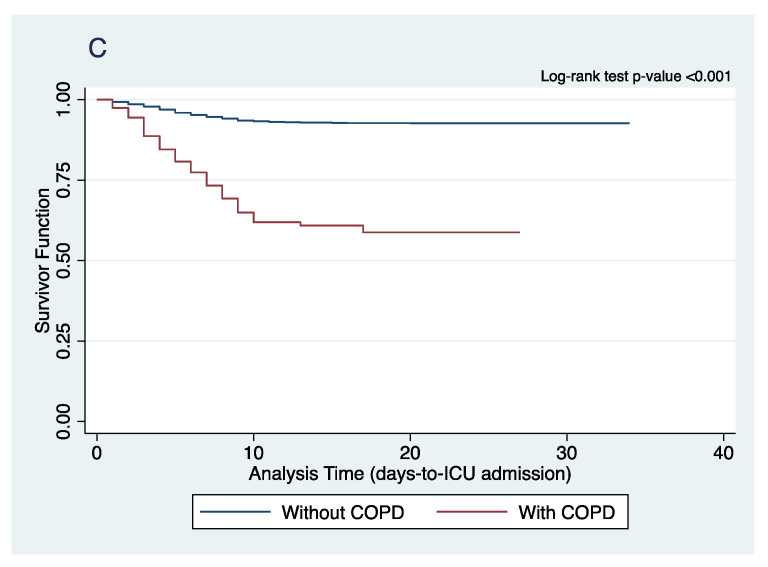
Comparison of Kaplan–Meier curves of (**A**) in-hospital mortality, (**B**) respiratory failure, and (**C**) ICU admission between COPD and non-COPD COVID-19 patients.

Table 2 summarizes the clinical outcomes of COVID-19 patients stratified according to presence of COPD diagnosis. COPD patients more commonly present with severe (38.1%, *p* < 0.001) or critical (33.6%, *p* < 0.001) COVID-19 compared to those without COPD. Figure 1 shows the Kaplan–Meier Curves on mortality, the development of respiratory failure and ICU admission. Our study showed that COVID-19 patients with COPD have higher rates of in-hospital mortality (Hazard ratio (HR) = 1.52, 95% CI (1.17, 1.97), *p* = 0.02), increased risk for developing respiratory failure predominantly from acute respiratory distress syndrome (HR = 2.90, 95% CI (2.24, 3.76), *p* < 0.001), and greater ICU admission rates (HR = 2.56, 95% CI (3.56, 4.17), *p* < 0.001), These suggests that COPD is a significant risk factor for mortality, the development of respiratory failure, and ICU admission for COVID-19 patients. 

On further analysis, however, there was no sufficient evidence to conclude any significant difference in time to the development of respiratory failure, the duration of mechanical ventilation, time to ICU admission, and the length of ICU stay between COPD and non-COPD patients. 

The association of having COPD diagnosis with different outcomes of interest in COVID-19 is shown in Table 3. Our COPD patients with COVID-19 were 2.0 times more likely to present as severe to critical COVID. They were also 1.7 times more likely to experience in-hospital mortality, 1.6 times more likely to have respiratory failure during the course of their admission and 2.0 times more likely to be admitted in the ICU. COPD COVID-19 patients have been found to also have 37% decreased odds of full/partial neurological improvement compared to patients without COPD. 

## 4. Discussion

Our study showed a small proportion of admitted COVID-19 patients had COPD diagnosis (1.4%). This was lower than the 7-11% reported in other studies [15,16]. This, however, does not mean that COPD patients are at a lower risk for contracting the disease. The cases in this study occurred during the early part of the pandemic when COVID-19 restrictions on mobility were heightened. Many of the COPD patients have been shielded from potential COVID-19 exposures because of these measures. The propensity to acquire COVID-19 among COPD patients in various studies have been confounded by these as well. Several reasons have been postulated as to the low prevalence of COPD among COVID-19 patients: usage of inhaled corticosteroids or bronchodilators, the already diseased state of the lungs may not be conducive for the SARS-CoV-2 to establish COVID-19, the presence of mucous plugs in large and small airways may hinder the virus from reaching the alveoli [17]. This under-representation may also be due to their pre-existing poor prognosis and decisions to continue with palliative care [18].

Our study highlighted that COPD patients who get admitted for COVID-19 have 3.4 times increased mortality and are 4.3 times likely to experience a severe or critical form of the disease that requires ventilatory support. This was also true in a study on 8395 patients, but not among patients with asthma, suggesting that the severity of the COVID-19 may be due to intrinsic immunological factors, such as type 2 inflammation [8]. Our study also showed increased health care utilization among COPD patients with COVID-19: 4.4 times likelihood of ICU admission, anti-inflammatory therapy (steroids and tocilizumab), antiviral, and antibacterial use. Furthermore, our study showed that COPD patients with COVID-19 have a higher incidence of neurologic symptoms and poor neurologic recovery. 

The reason behind the poor outcomes of COVID-19 among COPD patients continues to be investigated. Multiple factors have been postulated. First, COPD patients and smokers may be predisposed to SARS-CoV-2 infection. Studies have demonstrated that the gene expression for ACE-2 in bronchial epithelial cells from COPD patients is significantly elevated compared to control subjects [6,19]. This increased expression of the virus receptor may allow for the faster spread of the virus into the distal airways and alveoli, leading to progression from to a more severe COVID-19 pneumonia [20].

Another potential reason is that many COPD patients have poor lung function, small airway disease and emphysema resulting in significantly reduced respiratory functional reserve to cope with the intrapulmonary shunting created by a superimposed pneumonia or pulmonary vascular thromboembolic events observed in COVID-19 [19]. Increased intrapulmonary shunting has been associated with worse outcomes, including mortality [21].

Innate immune responses to viruses in COPD has been shown to be impaired as well. There are defective interferon responses to SARS-CoV-2 and this has been linked to an increased risk of severe COVID-19 [5]. COPD patients have colonizing pathogenic bacteria in their airways during the stable state. This colonization can cause secondary bacterial infections following respiratory viral infections. Bacterial infection is common among COVID-19 patients and leads to worse outcomes. This may be due to reduced antimicrobial responses during viral infections including decreased bacterial phagocytosis by alveolar macrophages and decreased antimicrobial peptide release [7].

The presence of comorbidities is also associated with more severe COVID-19 [5,9,10,11,22,23,24,25,26,27,28,29,30]. This was also seen in our patients and can potentially explain the poor outcomes.

The manifestations of COPD exacerbation and COVID-19 among COPD patients may be difficult to distinguish. A high index of suspicion and vigilance is recommended to ensure early diagnosis and access to life saving COVID-19 treatment modalities. 

Our study had several limitations. Our data collection did not differentiate different types of ventilatory support like invasive ventilation, high-flow nasal oxygen therapy, and non-invasive ventilation. As the diagnosis of COPD was based on medical history, there was no data on COPD severity, the use of maintenance medication and adherence, as well as lung function. These details may provide further insight into which subset or phenotype of COPD may have a poorer prognosis for COVID-19. Furthermore, our data on smoking did not differentiate current from previous smokers. It is interesting to know if smoking cessation has an impact on COVID-19 severity as well. We therefore recommend interested researchers to include these data in future research endeavors. 

## 5. Conclusions

This study on Filipino patients with COVID-19 supports the growing evidence that COPD patients developing COVID-19 are at higher risk mortality, at having severe and critical forms of COVID-19, at higher likelihood of needing ICU admission, and have higher chances of developing respiratory failure needing ventilatory support. 

## Figures and Tables

**Table 1:** Clinicodemographic characteristics stratified according to presence of COPD.

Features	All Patients	With COPD	Without COPD	*p* Value
	(*n* = 10,881)	(*n* = 156)	(*n* = 10,725)	
Socio-demographic data				
Age group, *n* (%)				<0.001
19–59 years	7047 (64.7)	33 (21.2%)	7014 (65.4%)	
≥60 years	3834 (35.2%)	123 (78.8%)	3711 (34.6%)	
Female, *n* (%)	5099 (46.9%)	25 (16.0%)	5074 (47.3%)	<0.001
Ever-smoker (past/current), *n* (%)	1026 (9.4%)	82 (52.5%)	944 (8.8%)	<0.001
Non-neurologic comorbidities, *n* (%)				
Hypertension	3647 (33.5%)	104 (66.7%)	3543 (33.0%)	<0.001
Diabetes mellitus	2191 (20.1%)	61 (39.1%)	2130 (19.8%)	<0.001
Cardiac disease ^a^	512 (4.7%)	29 (18.6%)	483 (4.5%)	<0.001
Chronic kidney disease	611 (5.6%)	17 (10.9%)	594 (5.5%)	0.004
Chronic liver disease	60 (0.6%)	2 (1.2%)	58 (0.5%)	0.212
Malignancy	244 (2.2%)	4 (2.5%)	240 (2.2%)	0.781
HIV/AIDS	37 (0.3%)	-	37 (0.3%)	1.000
Past neurologic history, *n* (%)				
Stroke/cerebrovascular	321 (3.0%)	10 (6.4%)	311 (2.9%)	0.026
Epilepsy	27 (0.3%)	-	27 (0.3%)	1.000
Neurodegenerative ^b^	44 (0.4%)	3 (1.9%)	41 (0.4%)	0.025
Headache syndrome	5 (0.1%)	-	5 (0.1%)	1.000
Demyelinating disorder	2 (0.0%)	-	2 (0.0%)	1.000
CNS Infection	5 (0.1%)	-	5 (0.1%)	1.000
PNS disorders ^c^	15 (0.1%)	-	15 (0.1%)	1.000
Respiratory and constitutional symptoms, *n* (%)				
Fever	3927 (36.1%)	80 (51.2%)	3847 (35.9%)	<0.001
Cough	4411 (40.5%)	114 (73.1%)	4297 (40.1%)	<0.001
Dyspnea	2703 (24.8%)	106 (67.9%)	2597 (24.2%)	<0.001
Rhinorrhea	607 (5.6%)	6 (3.9%)	601 (5.6%)	0.342
Sputum production	637 (5.9%)	23 (14.7%)	614 (5.7%)	<0.001
Sore throat	751 (6.9%)	9 (5.8%)	742 (6.9%)	0.574
Diarrhea	597 (5.5%)	5 (3.2%)	592 (5.5%)	0.208
Fatigue	713 (6.6%)	15 (9.6%)	698 (6.5%)	0.119
Others	1674 (15.3%)	16 (10.2%)	1658 (15.4%)	0.074
New onset neurological symptoms, *n* (%)				
Headache	607 (5.6%)	5 (3.2%)	602 (5.6%)	0.193
Nausea or vomiting	158 (1.5%)	2 (1.3%)	156 (1.5%)	1.000
Seizure	96 (0.9%)	1 (0.6%)	95 (0.9%)	1.000
Altered mental state ^d^	518 (4.7%)	13 (8.3%)	505 (4.7%)	0.035
Olfactory/taste dysfunction	663 (6.1%)	6 (3.9%)	657 (6.1%)	0.237
Dysfunctions of other senses ^e^	166 (1.5%)	1 (0.6%)	165 (1.5%)	0.735
Bulbar symptoms ^f^	122 (1.1%)	2 (1.3%)	120 (1.1%)	0.695
Motor symptoms	246 (2.2%)	5 (3.2%)	241 (2.3%)	0.406
Sensory symptoms	53 (0.5%)	1 (0.6%)	52 (0.4%)	0.536
Myalgia	256 (2.4%)	6 (3.9%)	250 (2.3%)	0.186
Others ^g^	33 (0.3%)	1 (0.6%)	32 (0.3%)	0.380
New-onset neurological disorders/complications, *n* (%)				
Encephalopathy ^h^	644 (5.9%)	19 (12.1%)	625 (5.8%)	0.001
Seizure	125 (1.2%)	1 (0.6%)	124 (1.1%)	1.000
Stroke/cerebrovascular ^i^	367 (3.4%)	9 (5.8%)	358 (3.3%)	0.095
CNS Infection ^j^	7 (0.07%)	-	7 (0.07%)	1.000
Others ^k^	14 (0.1%)	-	14 (0.1%)	1.000
Treatment/s received, *n* (%)				
Glucocorticoids	2844 (26.1%)	87 (55.8%)	2757 (25.7%)	<0.001
Tocilizumab	1029 (9.5%)	24 (15.3%)	1005 (9.4%)	0.011
Antiviral ^l^	1902 (17.4%)	46 (29.5%)	1856 (17.3%)	<0.001
Antibacterial	9014 (82.8%)	150 (96.2%)	8864 (82.7%)	<0.001
Others ^m^	3905 (35.9%)	75 (48.1%)	3830 (35.7%)	0.001

^a^ Includes heart failure, coronary artery disease, prior history of myocardial infarction, and other cardiac conditions. ^b^ Includes dementia, and movement disorders. ^c^ Includes peripheral nerve disease, neuromuscular junction disorder, and muscle disorder. ^d^ Includes altered sensorium, and confusion. ^e^ Includes visual, hearing, and vestibular dysfunctions. ^f^ Includes facial paresthesia, facial weakness, dysarthria, dysphonia, dysphagia, tongue weakness, and neck weakness. ^g^ Includes tremor, dystonia, choreoathetosis, bradykinesia, ataxia, and meningisimus. ^h^ Includes encephalopathy and anoxic brain injury. ^i^ Any acute cerebrovascular disease (no need to distinguish between infarction and hemorrhagic). ^j^ Includes encephalitis, meningitis, and meningoencephalitis. ^k^ Includes acute disseminated encephalomyelitis, optic neuritis, sensory ganglionitis, radiculitis, anterior horn syndrome, peripheral neuritis (Guillain Barre Syndrome and complications other than Guillain Barre Syndrome), neuromuscular disorder, and myositis. ^l^ Includes remdesivir, lopinavir, and ritonavir. ^m^ Includes chloroquine, hydroxychloroquine, convalescent plasma, and other therapies. COPD—Chronic obstructive pulmonary disease; HIV/AIDS—human immunodeficiency virus/acquired immunodeficiency syndrome; CNS—Central Nervous System; PNS—Peripheral Nervous System.

**Table 2:** Clinical outcomes of COVID-19 patients stratified according to COPD.

Outcomes	All Patients	With COPD	Without COPD	*p* Value
	(*n* = 10,881)	(*n* = 156)	(*n* = 10,725)	
COVID-19 severity at nadir, *n* (%)				<0.001
Mild/moderate	6690 (62.2%)	43 (28.3%)	6647 (62.7%)	
Severe	2354 (21.9%)	58 (38.1%)	2296 (21.7%)	
Critical	1707 (15.9%)	51 (33.6%)	1656 (15.6%)	
In-hospital mortality	1702 (15.6%)	60 (38.4%)	1642 (15.3%)	<0.001
Time to in-hospital mortality in days, median (IQR; range)	15 (13; 1 to 78)	20 (15.5; 2 to 65)	15 (13; 1 to 78)	0.004
Respiratory failure, *n* (%)	1608 (14.7%)	59 (37.8%)	1549 (14.4%)	<0.001
Cause/s ^a^				
Pneumonia	891 (55.4%)	30 (50.9%)	861 (55.6%)	0.472
ARDS	845 (52.6%)	43 (72.8%)	802 (51.7%)	0.001
Shock	140 (8.7%)	3 (5.1%)	137 (8.8%)	0.315
Central neurologic cause	86 (5.4%)	1 (1.7%)	85 (5.5%)	0.368
Pulmonary edema	32 (2.0%)	1 (1.7%)	31 (2.0%)	1.000
Pulmonary embolism	19 (1.1%)	2 (3.4%)	17 (1.1%)	0.152
Time to respiratory failure in days, median (IQR; range)	5 (4; 0 to 20)	6 (4; 1 to 13)	5 (4; 0 to 20)	0.342
Duration of invasive mechanical venticlation in days, median (IQR; range)	13 (12; 0 to 75)	15 (14; 1 to 62)	13 (12; 0 to 75)	0.150
Admitted to ICU, *n* (%)	1740 (15.99%)	70 (44.87%)	1670 (15.57%)	<0.001
Time to ICU admission in days, median (IQR; range)	5 (4; 0 to 20)	5 (5; 0 to 17)	5 (4; 0 to 20)	0.290
Length of ICU stay in days, median (IQR; range)	15 (11; 0 to 77)	15 (14; 1 to 64)	15 (11; 0 to 77)	0.923
Length of hospital stay ^b^ in days, median (IQR; range)	13 (9; 1 to 78)	14 (10; 2 to 65)	13 (9; 1 to 78)	0.003
Neurologic deficits, *n* (%)	2291 (21.0%)	35 (22.4%)	2256 (21.0%)	0.670
Neurologic outcome ^c^				<0.001
Full/partial improvement of neurologic deficits	1639 (88.0%)	12 (57.1%)	1627 (86.3%)	
No improvement of neurologic deficit	266 (13.9%)	9 (42.8%)	257 (13.6%)	

^a^ Not mutually exclusive. ^b^ Derived from overall length of stay for patients who were never admitted to the ICU; excludes length of ICU stay for those who were admitted in the ICU. ^c^ Patients with recorded data for neurologic outcome (*n* = 1905). COPD—Chronic obstructive pulmonary disease; COVID-19—coronavirus disease 2019; IQR—interquartile range; ICU—intensive care unit; ARDS—acute respiratory distress syndrome.

**Table 3:** Association of COPD with the different clinical outcomes.

Outcomes	Estimate ^a^	95% CI	*p* Value
Dichotomous outcomes			
Severe/critical COVID-19 at nadir ^b^	1.99	1.36, 2.91	<0.001
Neurologic deficit/s	0.64	0.41, 1.00	0.051
Full/partial improvement of neurologic deficit/s	0.37	0.14, 0.96	0.042
In-hospital mortality	1.66	1.17, 2.37	0.005
Respiratory failure	1.63	1.13, 2.34	0.009
ICU admission	2.02	1.41, 2.89	<0.001
Count outcomes			
Days of invasive mechanical ventilation	1.14	1.07, 1.21	<0.001
Days of ICU stay			
Among mild/moderate COVID-19	0.91	0.66, 1.24	0.547
Among severe COVID-19	1.03	0.93, 1.14	0.522
Among critical COVID-19	1.04	0.96, 1.12	0.318
Days of hospital stay			
Among mild/moderate COVID-19	0.95	0.88, 1.03	0.214
Among severe COVID-19	1.13	1.06, 1.20	<0.001
Among critical COVID-19	1.11	1.05, 1.19	0.001

^a^ Odds ratio for ordered and dichotomous outcomes, and incidence rate ratio for count outcomes; adjusted for age, sex, hypertension, diabetes mellitus, chronic cardiac disease, chronic kidney disease, cerebrovascular disease, demyelinating disease, and ever-smoker. ^b^ Dichotomized to mild/moderate, and severe/critical due to violation of the proportional odds assumption of ordinal logistic regression for the three-level severity, i.e., mild/moderate, severe, and critical. COPD—Chronic obstructive pulmonary disease; COVID-19—coronavirus disease 2019; ICU—intensive care unit; CI—confidence interval.

## Data Availability

Anonymized data not published within this article will be made available by request from any qualified investigator.
